# Gait Variability and Spatiotemporal Parameters During Emotion-Induced Walking: Assessment with Inertial Measurement Units

**DOI:** 10.3390/s25196222

**Published:** 2025-10-08

**Authors:** Marvin Alvarez, Angeloh Stout, Luke Fisanick, Chuan-Fa Tang, David George Wilson, Leslie Gray, Breanne Logan, Gu Eon Kang

**Affiliations:** 1 Department of Bioengineering, Erik Jonsson School of Engineering and Computer Science, The University of Texas at Dallas, Richardson, TX 75080, USA; marvin.alvarez@utdallas.edu (M.A.); angeloh.stout@utdallas.edu (A.S.); luke.fisanick@utdallas.edu (L.F.); 2 Department of Mathematical Sciences, School of Natural Sciences and Mathematics, The University of Texas at Dallas, Richardson, TX 75080, USA; chuan-fa.tang@utdallas.edu; 3 Department of Prosthetics-Orthotics, School of Health Professions, The University of Texas Southwestern Medical Center, Dallas, TX 75390, USA; 4 BrainRobotics, Austin, TX 78758, USA

**Keywords:** emotion, gait, gait variability, spatiotemporal parameters, inertial measurement units, wearable sensors

## Abstract

Emotion alters the way humans walk, yet most prior studies have relied on laboratory-based 3D motion capture systems. While accurate, these approaches limit translation to real-world settings and have largely focused on spatiotemporal parameters and joint motions. This study evaluated the feasibility of using inertial measurement units (IMUs) to detect emotion-related changes in gait variability as well as spatiotemporal gait parameters. Fourteen healthy young adults completed overground gait trials while wearing two ankle-mounted IMUs. Five target emotions, anger, sadness, neutral emotion, joy, and fear, were elicited using an autobiographical memory paradigm. The IMUs measured stride length, stride time, stride velocity, cadence, and gait variability. The results showed that stride length, stride time, stride velocity, and cadence significantly differed across emotions. Anger and joy were associated with longer strides and faster velocities, while sadness produced slower walking with longer stride times and reduced cadence. Interestingly, gait variability did not differ significantly across emotional states. These findings demonstrate that IMUs can capture emotion specific gait changes previously documented with motion capture, supporting their feasibility for use in natural and clinical contexts. This work advances understanding of how emotions shape gait and highlights the potential of wearable technology for unobtrusive emotion and mobility research.

## 1. Introduction

Emotions shape the way people move. Early research often described emotion-specific gait patterns using qualitative terms based on observations: for example, angry gaits are typically seen as heavy-footed, with long strides and vigorous arm swings, while happy gaits are described as energetic pace, extended strides, and pronounced arm movement. In contrast, sadness tends to slow the pace, resulting in shorter strides and reduced arm swing [1,2].

The effects of emotion on gait are evident not only in qualitative descriptions but also in quantitative measures. In particular, spatiotemporal and joint kinematic characteristics, often assessed with 3D motion capture technology, show systematic shifts with emotional states. For example, anger and joy are associated with approximately 20% faster gait, whereas sadness decreased gait speed by about 10%, all compared to neutral walking. These effects were accompanied by corresponding changes in stride length and cadence. The same study further showed larger sagittal ranges of motion across upper and lower limb joints during anger and joy, with especially pronounced effects in the upper limbs. Similar patterns have been reported across multiple studies [3,4,5].

In addition to spatiotemporal and joint kinematic changes, one interesting change in gait associated with emotion is the “tendency to act.” [6] This means that emotion not only influences the direction and energy behind our movements (e.g., speed, ranges of motion in upper limbs), but may also affect the underlying rhythmic coordination of the gait pattern [6]. Using 3D motion capture, it was reported that joy was associated with increased movement smoothness, as assessed by jerkiness (the first time derivative of acceleration) normalized to movement time and distance in the whole-body center of mass, as well as in upper and lower extremity joints, compared to sadness, which resulted in jerkier movement and poorer coordination [7]. Furthermore, another previous study showed that emotions alter intersegmental coordination, i.e., the dynamic relationships between limb segments, beyond what would be expected from changes in speed alone [8]. Their findings indicate that the kinematic characteristics of different emotional states include changes in amplitude, sinusoidality, and phase relationships between limb movements, all of which contribute to the overall smoothness and expressiveness of gait. Taken together, these studies support the idea that the tendency to act under emotion is not just about movement vigor, but is embodied in the quality, smoothness, and coordination of human gait.

Given the well-established influences of emotion on gait speed and coordination, it is notable that no studies have directly examined how emotions affect gait variability, which refers to the stride-to-stride fluctuations in walking. Gait variability is a sensitive indicator of how the nervous system regulates movement, showing the adaptability and stability of locomotor control [9]. Emerging evidence shows that variability can reveal subtle disruptions or adaptations in gait patterns that may not be captured by average measures alone (e.g., gait speed, stride length) [10]. For instance, excessive gait variability has been linked to an increased risk of falls and can signal neuromotor dysfunction even when average gait speed appears normal. In the context of emotion, examining gait variability could provide novel information into how emotional states destabilize or modulate the fine-tuning of movement, potentially discovering changes in underlying sensorimotor processing that extend beyond visible changes in speed or range of motion. By capturing these dynamic fluctuations in gait, researchers may better understand the ways in which emotion shapes movement, opening the door to new clinical and scientific applications in emotional gait assessment.

In addition to gait variability, another important gap in emotional gait research is the choice of tools used to assess gait. To date, most quantitative studies have relied heavily on 3D motion capture, likely because of its precision and long-standing role in research [3,7,8,11,12,13]. While 3D motion capture provides high accuracy and has been central to important scientific discoveries linking emotion and gait, there are clear limitations in terms of practicality, translation, and application. Many studies on emotion and gait hold promise for early detection of emotional disorders such as depression, bipolar disorder, or agitation, but despite its accuracy [14], 3D motion capture is unsuitable for such clinical or real-world uses. Beyond cameras, commonly used alternatives include instrumented walkways (spatiotemporal timing across embedded sensors), force plates (ground-reaction forces and loading symmetry), and pressure-sensing insoles (plantar pressure and center-of-pressure trajectories). These systems can quantify key gait features but are often fixed-site, costly, or task-limited. Among available motion analysis tools, inertial measurement units (IMUs) may help address this gap. Compared with 3D motion capture or instrumented walkways, IMUs are substantially less expensive, portable, and widely adapted in gait research beyond the emotion domain. Recent studies suggest that IMUs provide reliable estimates of spatiotemporal parameters such as gait speed, stride length, cadence, and gait variability [15,16]. In addition, contemporary IMU pipelines can reconstruct lower limb joint kinematics when paired with robust sensor to body calibration procedures that combine anatomical alignment with functional movements [17]. Given these advantages, IMUs appear to be a strong candidate for expanding the applicability of emotion and gait research beyond the laboratory. Despite this potential, there remains little documented work applying IMUs specifically to emotional gait.

The purpose of this study was to determine the extent to which emotional states influence spatiotemporal gait parameters and gait variability using IMUs. We hypothesized that emotional states would significantly affect these gait variables. The novelty of this study lies in applying IMUs to emotion-induced walking to (i) replicate emotion-linked spatiotemporal changes previously reported with emotion capture only, and (ii) directly evaluate whether stride-to-stride gait variability is modulated by emotion in healthy young adults, an issue that, to our knowledge, has not been examined with any methodology.

## 2. Materials and Methods

### 2.1. Participants

We recruited a convenience sample of 14 recreationally active, healthy young adults from the University of Texas at Dallas community. All participants had no history of neurological disorders (e.g., stroke) or musculoskeletal conditions (e.g., prior knee injury). Our sample size was consistent with previous emotion–gait studies [7,8,11]. We focused on healthy young adults to minimize potential confounding effects on emotional responses and gait patterns. Informed consent, approved by the University of Texas at Dallas Institutional Review Board, was obtained from all participants prior to participation. Following consent, demographic information including age, height, weight, sex, and physical activity level was collected. Physical activity was assessed using the International Physical Activity Questionnaire (IPAQ) short form to confirm that participants were recreationally active [18].

### 2.2. Emotion Induction Preparation

We used an autobiographical memory paradigm to induce five target emotions: anger, sadness, neutral, joy, and fear. This paradigm has been widely used in both emotion–gait and broader emotion research, and it has been shown to reliably elicit emotional states [3,7,11,19]. We selected these five emotions based on prior studies, the emotion circumplex model, and our own research experience [3,7,8,11,19].

As part of the autobiographical memory task, after providing consent and completing demographic and clinical assessments, participants were asked to write a personal memory for each of the five target emotions using a structured worksheet (Appendix A). Prompts were provided to help elicit vivid memories, for example, *“Think of a time in your life when you felt very offended, furious, enraged, or wanted to explode”* for anger; *“Think of a time when you felt in despair, very low or depressed, or wanted to withdraw from the world”* for sadness; *“Think of a time when you felt exhilarated, euphoric, or very playful, or wanted to jump up and down”* for joy; *“Think of a time when you felt scared, fearful, or terrified, or as though you were in danger”* for fear; and *“Think of a time when you did not feel any particular emotion, such as putting gas in your car or doing your laundry”* for neutral. The memories written in this worksheet were later used to help participants recall and re-experience the target emotions during the gait trials.

### 2.3. Experimental Procedures

**Figure 1:**
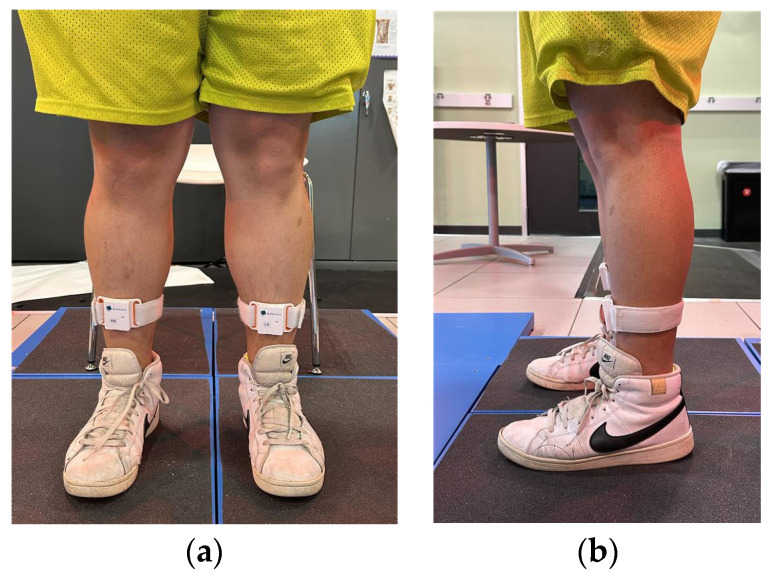
Attachment of IMUs at the distal tibia, just superior to the malleoli: (**a**) the front view and (**b**) the side view.

After completing the autobiographical memory worksheet, participants were prepared for the gait trials. We first provided a briefing, explaining that they would be asked to perform walking trials while recalling one of the target emotions and wearing IMUs. Following the briefing, two IMUs (BioSensics, Newton, MA, USA) were attached to the distal tibia, just superior to the malleoli, as shown in Figure 1. This placement was selected to optimize shank angular-velocity signals for gait-event detection and stride timing and is consistent with validated protocols for estimating spatiotemporal parameters and gait variability [20,21,22]. Bilateral shank placement was used to capture events on both limbs for stride-based outcomes while maintaining a minimal, field-feasible setup. Each IMU contained a tri-axial accelerometer and a tri-axial gyroscope, enabling the collection of linear acceleration and angular velocity of the shanks at a sampling rate of 100 Hz.

Participants performed gait trials on a 30 ft walkway in the Neuromuscular and Musculoskeletal Biomechanics Laboratory at the University of Texas at Dallas. They were asked to walk across the walkway while recalling one of the target emotions. To induce these emotions, participants stood at one end of the walkway and were handed their completed autobiographical memory worksheet. A target emotion (e.g., joy) was selected by us randomly, and participants were asked to read their written memory to recall the associated feeling. After reading, the worksheet was collected, and participants were encouraged to remain focused on the memory and to fully immerse themselves in the emotional state. Verbal encouragement was also provided (e.g., “be in the memory”). Participants were instructed to begin walking once they strongly felt the target emotion and were allowed as much time as needed for induction.

Following each gait trial, participants rated the intensity of the target emotion as well as other non-target emotions using a 5-point Likert scale (0 = not at all, 1 = a little bit, 2 = moderately, 3 = quite a bit, 4 = extremely). This was recorded using the feeling questionnaire (Appendix B). Each target emotion was tested in a block of three gait trials, after which participants moved on to the next target emotion. Between emotion blocks, a one-minute washout task, such as card sorting or rearranging a Rubik’s cube, was completed to minimize carryover effects.

### 2.4. Data Analysis

We excluded gait trials in which participants rated intensity ratings of *not at all* or *a little bit* for the target emotion when the trial involved anger, sadness, joy, or fear. For the neutral condition, trials were included only if participants rated *not at all* or *a little bit* for all non-neutral emotions.

Gait parameters were generated by the LEGSys software (BioSensics) using its embedded, validated algorithms. Initial contact and toe-off were identified from characteristic features of the sagittal shank angular-velocity signal acquired by the gyroscopes; stride time was computed as the interval between successive initial contacts of the same limb; cadence was calculated as steps per minute; stride length was estimated using an inverted-pendulum model parameterized by limb length (derived from participant height); and stride velocity was obtained as stride length divided by stride time. For each trial, the system provided a stride-by-stride time series; we computed the trial mean for each variable and gait variability as percent coefficient of variation (%CV = 100 × SD/mean) across strides. No additional filtering or manipulation of the raw signals was performed beyond the manufacturer’s default signal conditioning. Algorithmic details and validation of this approach for shank-mounted IMUs are reported in [20,21].

All analyses used a within-subject framework with emotion as a five-level repeated factor for each gait variable. Descriptive statistics were calculated for each emotion and expressed as mean ± standard deviation. Normality of outcome measures was tested using the Shapiro–Wilk test. When assumptions were met, a one-way repeated measures analysis of variance (ANOVA) was conducted; for data not normally distributed, the Friedman test was applied instead. Effect sizes were calculated using Kendall’s W or partial eta squared, interpreted as small (0.1–0.3), medium (0.3–0.5), or large (>0.5), with 95% confidence intervals reported. Pairwise post hoc analyses were conducted using either the Wilcoxon signed-rank test or the paired t-test, depending on normality. Effect sizes for post hoc comparisons were reported as r or Cohen’s dz, also with 95% confidence intervals. To account for the large number of variables and reduce the risk of false positives, *p*-values were adjusted for multiple comparisons using the Benjamini–Hochberg false discovery rate (FDR) procedure, applied to both the omnibus (Friedman/ANOVA) and post hoc analyses. Statistical significance was set at *p* < 0.05. All statistical analyses were performed in RStudio (Version 4.4.1).

## 3. Results

### 3.1. Participant Characteristics and Emotion Manipulation Check

Participant demographic and physical activity level data are presented in Table 1. In total, 210 emotional gait trials were collected (14 participants × 5 target emotions × 3 repetitions). Of these, 17 trials from 6 participants were excluded due to low self-reported intensity of the target emotion. This resulted in 193 trials being included in the analysis: 40 Angry, 39 Sad, 42 Neutral, 38 Joy, and 34 Fear.

Mean intensity ratings for the target emotions are presented in Table 2. As expected, participants reported the highest intensity ratings for the target emotion in each trial (e.g., anger during Angry trials, sadness during Sad trials), while ratings for non-target emotions remained low. Based on self-report, the target emotion was rated higher than non-targets in the corresponding trials, indicating limited overlap among emotional states.

### 3.2. Spatiotemporal Gait Parameters

**Figure 2:**
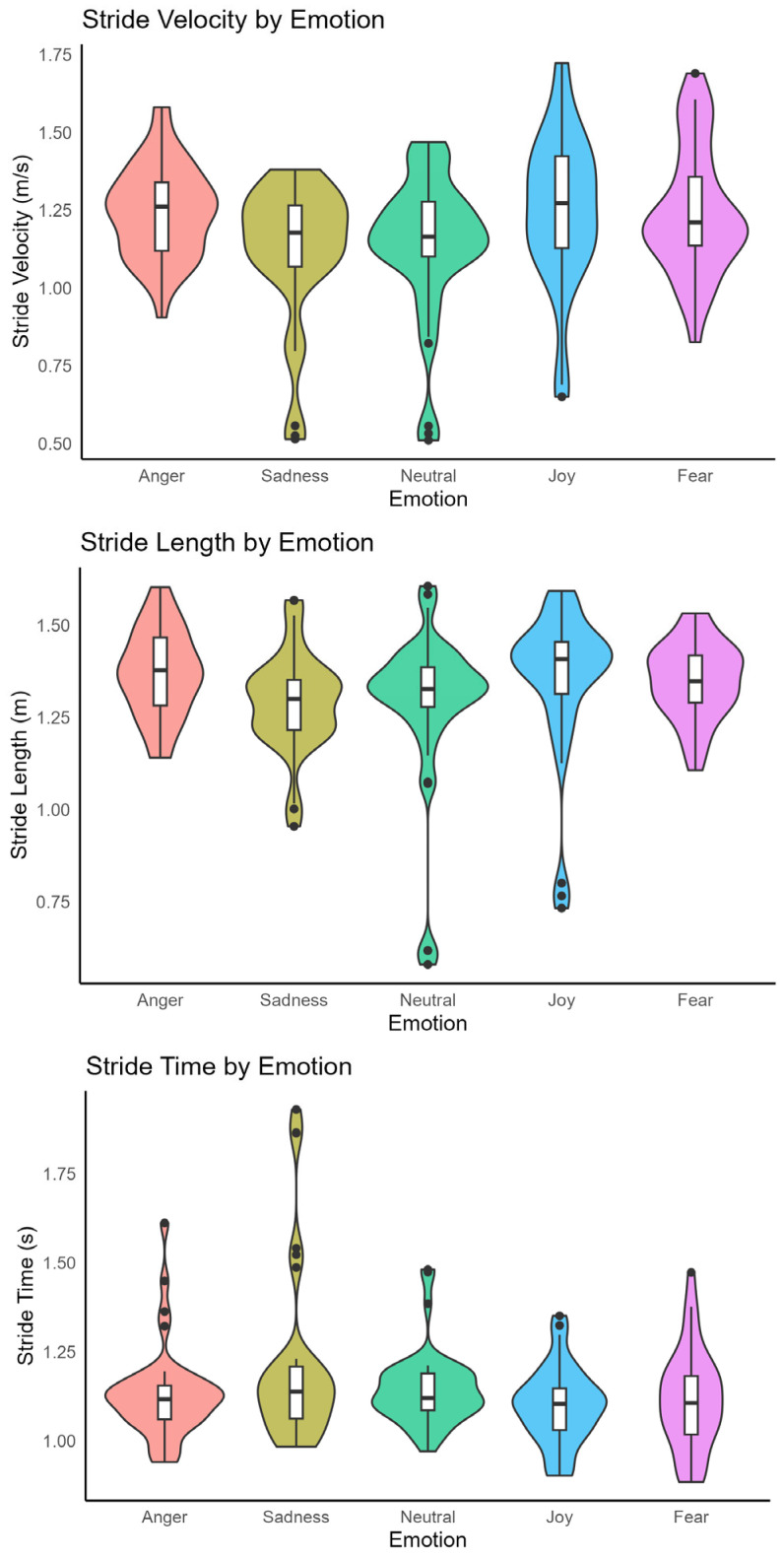
Violin plots of gait variables by emotion: (**Top**) stride velocity (m/s) (**Middle**) stride length (m), and (**Bottom**) stride time (s) for Angry, Sad, Neutral, Joy, and Fear conditions. The violin width reflects the distribution of observations; the central white box shows the interquartile range with the median line, and whiskers indicate 1.5 × interquartile range. Overlaid points are individual trial values.

We presented stride length, stride time, and stride velocity across emotions in Figure 2. Emotion significantly changed all three gait variables (*p* < 0.01). Stride length was greater in the angry (1.37 ± 0.03 m), joyful (1.35 ± 0.05 m), sad (1.30 ± 0.03 m), and fearful (1.35 ± 0.03 m) conditions compared to neutral (1.29 ± 0.06 m), with a significant difference observed between sadness and joy, neutral and joy, anger and sadness, and anger and neutral (all *p* < 0.05, r between 0.54 and 0.84).

Stride time was longest during sad walking (1.20 ± 0.06 s) and shortest during joyful walking (1.10 ± 0.03 s), with sad differing significantly from both joyful (1.10 ± 0.11 s), neutral (1.14 ± 0.11 s), and fearful (1.13 ± 0.15 s) walking. Pairwise comparison showed significant differences between sadness and joy, and neutral and joy (all *p* < 0.05, r = 1.03 and 0.79, respectively).

Stride velocity was higher in angry (1.24 ± 0.04 m/s), joyful (1.25 ± 0.06 m/s), and fearful (1.22 ± 0.05 m/s) walking compared to neutral (1.14 ± 0.06 m/s), while sad walking was slower (1.12 ± 0.06 m/s). A significant pairwise differences were found between neutral and joy, anger and neutral, and anger and sad (all *p* < 0.05, r = 0.81, 0.68, and 0.66, respectively). There was marginal pairwise difference between sad and joy (*p* = 0.05, r = 0.62).

The other spatiotemporal parameters for each target emotion are presented in Table 3. Among these, cadence showed significant differences across emotions (*p* < 0.05). In contrast, swing and stance phases, as well as double support phases, did not differ significantly among the target emotions.

### 3.3. Gait Variability

**Figure 3:**
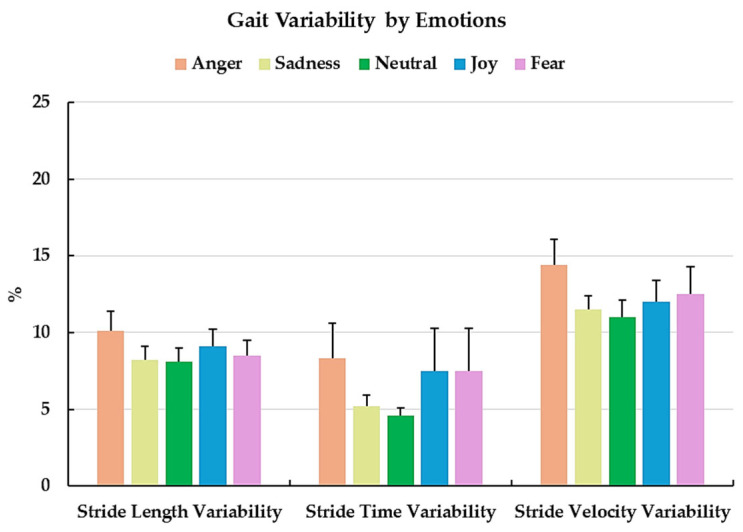
Mean and standard error of gait variability (%) in stride length, stride time, and stride velocity across target emotions (angry, sad, neutral, joyful, and fearful).

Gait variability in stride length, stride time, and stride velocity did not differ significantly across the target emotions, with small effect sizes (Figure 3). For stride length variability, angry (10.1 ± 1.3%), sad (8.2 ± 0.9%), joyful (9.1 ± 1.1%), and fearful (8.5 ± 1.0%) trials were all slightly higher than neutral (8.1 ± 0.9%). Stride time variability showed a similar trend, with angry (8.3 ± 2.3%), sad (5.2 ± 0.7%), and fearful (7.5 ± 2.8%) trials exceeding neutral (4.6 ± 0.5%), while joyful walking (7.5 ± 2.8%) remained comparable. For stride velocity variability, the values were higher during angry (14.4 ± 1.7%), sad (11.5 ± 0.9%), joyful (12.0 ± 1.4%), and fearful (12.5 ± 1.8%) trials compared to neutral (11.0 ± 1.1%).

## 4. Discussion

In this study, we examined the effects of emotional states on spatiotemporal gait parameters and gait variability using IMUs. The novelty of this work lies in two aspects: first, the investigation of gait variability under different emotional states, and second, the use of IMUs as the measurement tool. We found that emotional states significantly influenced stride length, stride time, stride velocity, and cadence, while swing, stance, and double support phases were largely unaffected. Gait variability did not differ significantly across emotions, although non-neutral conditions showed a small, non-significant increase relative to neutral. These findings provide evidence that IMUs can detect emotion-related changes in spatiotemporal parameters, supporting their feasibility for emotional gait research.

Beyond reproducing established motion-capture findings with a wearable modality, our design directly tests whether emotion modulates stride-to-stride variability during overground walking, thereby linking emotion–gait relationships to a body-worn platform suitable for real-world assessment. By using a low-burden, bilateral shank IMU setup, we also demonstrate a measurement pathway that lab-based technologies cannot readily provide, namely, ecologically valid acquisition during naturalistic tasks, repeatable induction outside the lab, and the potential for longitudinal or in-home monitoring. Together, these contributions move the field from laboratory demonstrations toward translational, wearable assessment of emotion–gait relationships.

Our IMU-derived spatiotemporal effects were in line with those reported with 3D motion capture: joy and anger increased gait speed, stride length, and cadence, whereas sadness decreased gait speed, stride length, and cadence, consistent with prior laboratory studies [7,11]. This consistency supports the criterion validity of a two-sensor, shank-mounted IMU configuration for detecting emotion-linked changes in stride length, stride time, stride velocity, and cadence under overground conditions. At the same time, IMUs and cameras may offer complementary capabilities. For example, cameras provide precise whole-body joint kinematics and segment coordination but are lab-based. IMUs are more portable, and potentially more scalable for naturalistic or longitudinal monitoring. IMUs also offer spatiotemporal parameters and (with additional IMUs) segment/joint kinematics. IMUs also rely on embedded algorithms (gait-event detection, inverted-pendulum models) rather than direct 3D reconstruction, which may limit access to upper-body or intersegmental features commonly examined in camera-based work. Thus, our results both corroborate motion capture findings at the spatiotemporal level and delineate practical trade-offs: IMUs enable ecologically valid assessments but do not yet replace full-body kinematic analyses in emotion–gait research.

An interesting finding from this study was the robust effect of sadness and joy on gait patterns. Sad walking was consistently slower, with longer stride times and reduced cadence, reflecting a less dynamic gait profile [11,19]. In contrast, joyful walking was characterized by shorter stride times, longer stride lengths, and faster velocities, indicating a more energetic and coordinated movement pattern. Notably, angry and fearful walking also showed elevated stride velocity and stride length compared to neutral, suggesting that high-arousal negative emotions may share certain locomotor features with joy.

However, despite these significant differences in several spatiotemporal parameters, gait variability did not differ significantly across the target emotions, which did not support our hypothesis. This pattern indicates that emotional states altered the magnitude and tempo of walking, for example faster or slower steps and longer or shorter strides, without degrading stride-to-stride regularity in this cohort. A likely explanation is that recreationally active, healthy young adults maintain stable locomotor control across emotional conditions, preserving step-to-step consistency even when average speed or stride length changes. Methodological factors may also contribute, including the short overground distance that yields relatively few strides per trial. Taken together, these results delineate a boundary condition for emotion effects on variability and suggest that emotion-related changes in coordination and smoothness may be more evident with longer walking bouts, additional sensors on the trunk and upper limbs, or in populations with reduced motor stability, in line with prior work on smoothness and intersegmental relations [7,8].

In addition to robust motor control in young, active adults, several methodological and measurement factors could have attenuated emotion effects on gait variability. The short-overground distance may have possibly yielded few strides per trial, reducing precision of gait variability [23,24]. Second, ankle-mounted IMUs are optimized for gait-event timing and spatiotemporal means, whereas subtle emotion effects may emerge more strongly in upper-body sway or multi-segment coordination rather than in stride-interval dispersion. Third, the variability metric used here (percent coefficient of variation) captures linear dispersion; complementary nonlinear metrics (for example, sample entropy or detrended fluctuation analysis) may detect changes in temporal structure. These considerations motivate designs with longer walking bouts, additional sensors on the trunk and upper limbs, and a broader suite of variability and complexity measures.

From a scientific perspective, our study extends emotional gait research by showing that IMUs can effectively capture spatiotemporal gait changes that have traditionally required 3D motion capture. This opens a promising path for expanding research beyond controlled laboratory environments, where portability and cost-effectiveness are crucial. For example, emotional gait research could be conducted in home or community settings [25,26], where participants may experience a more natural emotional feeling compared to the often artificial atmosphere of the lab with reflective markers and strict protocols.

Clinically, these findings may inform future work on unobtrusive behavioral markers of emotional disorders such as depression or anxiety, but additional evidence is required before clinical application [14,27]. For instance, the slowing of gait and lengthening of stride time observed in sadness could serve as candidate indicators of psychomotor retardation, and fast velocities during anger or fear could reflect heightened arousal, yet these possibilities require validation in clinical cohorts. Similarly, fear of falling is an emotional state that affects gait and quality of life [28], but whether IMU-based measures **can** identify individuals at risk remains to be tested. At present, IMU assessments should be viewed as research tools that might complement conventional evaluations pending further validation.

Limitations should be acknowledged. First, the sample size was modest (n = 14), and together with homogeneous, recreationally active young cohort, likely limited statistical power to detect small effects, particularly for gait variability. An a priori power check suggests that detecting a small to moderate within-subject effect in variability would require approximately n = 30 for the omnibus repeated measures test with five emotions at α = 0.05 and power = 0.80, assuming a within-subject correlation of 0.50. Homogeneity can reduce between-subject dispersion, further lowering sensitivity. Future studies should enroll larger and more diverse samples, report effect sizes and confidence intervals to contextualize estimates, and conduct a priori power analyses tailored to variability outcomes. Second, the participants were healthy young adults, limiting generalizability to older adults or clinical populations where gait variability may be more sensitive to emotional states. Third, the autobiographical memory paradigm was validated only by self-report ratings. We did not collect concurrent physiological or behavioral indices of emotional state in this pilot, which limits verification of the induction beyond subjective reports. Future work should incorporate multimodal validation (e.g., heart rate variability, respiration) and behavioral markers such as facial affect coding or voice features to strengthen the manipulation check. Finally, gait was measured over a 30 ft walkway, which may not fully capture longer-term walking dynamics.

Future research should address these limitations by including larger and more diverse samples, particularly older adults and clinical groups, to determine whether emotional influences on gait variability are more pronounced in populations with reduced motor stability [29]. In such populations, including older adults and individuals with neurological or affective disorders, gait variability is typically elevated [30,31,32]. Under these conditions, emotional states, especially negative or high-arousal emotions such as fear, may more strongly disrupt gait pattern than in healthy young adults, yielding larger and more detectable variability effects. Accordingly, studies that recruit older adults and clinical cohorts are well positioned to test whether emotion-related changes in variability are amplified relative to the present sample. Longitudinal designs could also explore whether emotion-related gait changes predict mental health outcomes, such as relapse in depression or severity of anxiety. In addition, incorporating multimodal assessments, including physiological measures (e.g., heart rate variability) and neural recordings (e.g., electroencephalography), alongside gait metrics may offer a more comprehensive understanding of how emotions modulate motor control. Beyond walking, other movements such as sit-to-stand transitions or gait initiation could provide further insight into the interaction between emotion and motor behavior [33]. Finally, extending this work into real-world environments using wearable IMUs would strengthen clinical applications, enabling continuous and unobtrusive monitoring of emotional states in daily life.

In conclusion, this study demonstrated that emotional states significantly influence stride length, stride time, stride velocity, and cadence, while having limited effects on swing, stance, and double support phases. Contrary to expectations, gait variability did not significantly differ across emotional states, suggesting that stride-to-stride consistency may be preserved even when gait speed and rhythm change with emotion. Importantly, this study highlights the feasibility of using IMUs to capture emotion-related gait changes, paving the way for portable and clinically relevant applications in emotional gait research.

## Figures and Tables

**Table 1:** Demographic characteristics.

Measures	Outcomes (mean ± std)
Demographic	
N	14 (number of females = 9)
Age, years	20.3 ± 2.2
Height, m	1.7 ± 0.1
Weight, kg	63.6 ± 8.3
Body-Mass Index, kg/m^2^	22.8 ± 2.6
Physical Activity Level	
Vigorous Activity (Days)	3.1 ± 2.2
Vigorous (Minutes)	55.7 ± 54.1
Moderate (Days)	2.1 ± 2.4
Moderate (Minutes)	34.3 ± 42.2
Walking (Days)	4.5 ± 2.8
Walking (Minutes)	84.3 ± 91.3
Sitting (Hours per Day)	5.7 ± 2.6
Sitting (Minutes per day)	342.9 ± 153.7

Note: STD = standard deviation; IPAQ = International Physical Activity Questionnaire.

**Table 2:** Emotion intensity across gait trials.

Target Emotions	Mood Intensity				
	Angry	Sad	Joyful	Fearful	Neutral
Angry	2.8 ± 0.7	0.6 ± 1.0	0.0 ± 0.0	0.5 ± 0.9	0.0 ± 0.0
Sad	0.9 ± 1.2	3.2 ± 0.7	0.1 ± 0.4	0.9 ± 1.3	0.1 ± 0.3
Joyful	0.2 ± 0.6	0.1 ± 0.3	3.3 ± 0.7	0.0 ± 0.2	0.3 ± 0.6
Fearful	0.3 ± 0.7	0.9 ± 1.0	0.0 ± 0.2	3.0 ± 0.7	0.0 ± 0.2
Neutral	0.1 ± 0.4	0.3 ± 0.5	0.2 ± 0.4	0.1 ± 0.4	3.2 ± 1.2

Note: Values represent mean ± standard deviation.

**Table 3:** Cadence (steps/min), swing and stance phases (%), and double limb support (DLS; %) across target emotions.

	Angry	Sad	Neutral	Joyful	Fearful	*p*-Value	Adjusted *p*	Effect Size (95% CI)
Cadence	108.7 ± 8.7	103.1 ± 15.3	105.8 ± 8.8 ^A^*	110.3 ± 10.5 ^S^*	108.4 ± 13.0	0.0015	0.002	W = 0.31 (0.23, 0.55)
Swing (Left)	40.3 ± 2.2	39.5 ± 3.0	40.1 ± 2.4	40.3 ± 1.9	40.0 ± 2.4	0.480	0.554	W = 0.06 (0.02, 0.30)
Swing (Right)	41.2 ± 1.5	40.6 ± 2.7	41.0 ± 1.5	41.3 ± 1.6	41.4 ± 1.5	0.183	0.343	W = 0.11 (0.03, 0.45)
Stance (Left)	59.7 ± 2.2	60.5 ± 3.0	59.9 ± 2.4	59.7 ± 1.9	60.0 ± 2.4	0.480	0.554	W = 0.06 (0.02, 0.29)
Stance (Right)	58.8 ± 1.5	59.4 ± 2.7	59.0 ± 1.5	58.7 ± 1.6	58.6 ± 1.5	0.183	0.342	W = 0.11 (0.03, 0.42)
DLS (Initial Right)	10.0 ± 2.7	10.5 ± 3.4	10.2 ± 2.3	9.8 ± 2.1	9.9 ± 2.4	0.878	0.878	W = 0.02 (0.01, 0.24)
DLS (Terminal Left)	8.4 ± 1.4	9.4 ± 2.2	8.6 ± 1.8	8.6 ± 1.5	8.7 ± 1.6	0.063	0.410	η^2^p = 0.16 ([0.05, 0.41)

Note: Values represent mean ± standard deviation. Superscript letters indicate significant differences between target emotions based on pairwise comparisons: A = Angry, S = Sad, * *p* < 0.05.

## Data Availability

Data is not available due to institutional restrictions.

## Appendix A

### Autobiographical Memories Worksheet

Later in this study, we will ask you to visualize and try to relive specific memories from your own life experience. To facilitate this process, we would like you to locate these specific memories right now, by answering a few questions about each. Take a moment to consider each of the following five autobiographical memories.

NOTE: Identify people with initials and relationship descriptions only (e.g., my mom, my boyfriend, T.S.).

MEMORY #1

Think of a time in your life when you felt very offended, for instance, when you felt furious or enraged, or felt like you wanted to explode.

Using only a few words, please indicate:

(a) … Where you were:
(b) … Who you were with:
(c) … What caused the feeling/what was it about?

MEMORY #2

Think of a time in your life when you felt in despair, for instance, when you felt low or depressed, or felt like you wanted to withdraw from the world.

Using only a few words, please indicate:

(a) … Where you were:
(b) … Who you were with:
(c) … What caused the feeling/what was it about?

MEMORY #3

Think of a time in your life when you did not feel any emotion, for instance, when you put gas in your car or you did laundry.

Using only a few words, please indicate:

(a) … Where you were:
(b) … Who you were with:
(c) … What caused the feeling/what was it about?

MEMORY #4

Think of a time in your life when you felt exhilarated, for instance, when you felt euphoric or very playful, or felt like you wanted to jump up and down.

Using only a few words, please indicate:

(a) … Where you were:
(b) … Who you were with:
(c) … What caused the feeling/what was it about?

MEMORY #5

Think of a time in your life when you felt scared, for instance, when you felt fearful or terrified, or felt like you were in danger.

Using only a few words, please indicate:

(a) … Where you were:
(b) … Who you were with:
(c) … What caused the feeling/what was it about?

## Appendix B

### Feeling Questionnaire

Instructions: In any given circumstance, people often have a number of different feelings. Please think back to how you felt while you were moving. Please indicate how much of emotion you felt during that time. Use the following 0 to 4 scale to make your ratings.

0 = Not at all

1 = A little bit

2 = Moderately

3 = Quite a bit

4 = Extremely

_______ I felt angry, irritated, annoyed.

_______ I felt glad, happy, joyful.

_______ I felt sad, downhearted, unhappy.

_______ I felt scared, fearful, afraid.
